# Accumulation of dipeptide repeat proteins predates that of TDP‐43 in frontotemporal lobar degeneration associated with hexanucleotide repeat expansions in C9ORF72 gene

**DOI:** 10.1111/nan.12178

**Published:** 2015-04-30

**Authors:** Atik Baborie, Timothy D. Griffiths, Evelyn Jaros, Robert Perry, Ian G. McKeith, David J. Burn, Masami Masuda‐Suzukake, Masato Hasegawa, Sara Rollinson, Stuart Pickering‐Brown, Andrew C. Robinson, Yvonne S. Davidson, David M. A. Mann

**Affiliations:** ^1^ Department of NeuropathologyWalton Centre for Neurology and NeurosurgeryLiverpoolUK; ^2^ Institute of NeuroscienceNewcastle University Medical SchoolNewcastle upon TyneUK; ^3^ Neuropathology/Cellular PathologyRoyal Victoria InfirmaryNewcastle upon TyneUK; ^4^ Institute for Ageing and HealthNewcastle University, Campus for Ageing and VitalityNewcastle upon TyneUK; ^5^ Institute of Brain, Behaviour and Mental HealthUniversity of ManchesterManchesterUK; ^6^ Institute of Brain, Behaviour and Mental HealthUniversity of Manchester, Salford Royal HospitalSalfordUK; ^7^ Department of Neuropathology and Cell BiologyTokyo Metropolitan Institute of Medical ScienceTokyoJapan

**Keywords:** C9ORF72, dipeptide repeat proteins, frontotemporal lobar degeneration, hexanucleotide repeat expansion

## Abstract

**Aims:**

Frontotemporal lobar degeneration (FTLD) and motor neurone disease are linked by the possession of a hexanucleotide repeat expansion in C9ORF72, and both show neuronal cytoplasmic inclusions within cerebellar and hippocampal neurones which are TDP‐43 negative but immunoreactive for p62 and dipeptide repeat proteins (DPR), these being generated by a non‐ATG RAN translation of the expanded region of the gene.

**Methods:**

Twenty‐two cases of FTLD from Newcastle were analysed for an expansion in C9ORF72 by repeat primed PCR and Southern blot. Detailed case note analysis was performed, and blinded retrospective clinical impressions were achieved by review of clinical histories. Sections from all major brain regions were immunostained for TDP‐43, p62 and DPR. The extent of TDP‐43 and DPR pathology in expansion bearers was compared with that in 13 other previously identified cases from the Manchester Brain Bank with established disease.

**Results:**

Three Newcastle patients bearing an expansion in C9ORF72 were identified. These three patients died prematurely, two from bronchopneumonia within 10 months and 3 years of onset, and one from myocardial infarction 3 years after onset. In all three, DPR were plentiful throughout all cerebral cortical regions, hippocampus and cerebellum, but TDP‐43 pathological changes were sparse. The severity of DPR pathological changes in these three patients was similar to that in the Manchester series, although the extent of TDP‐43 pathology was significantly less.

**Conclusion:**

Widespread accumulation of DPR within nerve cells may occur much earlier than that of TDP‐43 in patients with FTLD bearing expansion in C9ORF72.

## Introduction

Frontotemporal lobar degeneration (FTLD) presents with a spectrum of clinical entities [1, 2] associated with various patterns of pathological protein accumulation [3]. A hexanucleotide repeat expansion in intron 1 of the chromosome 9 open reading frame 72 (*C9ORF72*) gene is the major genetic factor so far known to be associated with both FTLD and motor neurone disease (MND) [4, 5, 6]. A pathological accumulation of p62 protein, as neuronal cytoplasmic inclusions (NCI), within granule cells of the cerebellum, and CA4 neurones of the hippocampus has been claimed to be a distinctive histological feature of this particular genetic form of FTLD [7, 8, 9]. P62 is a marker for proteins destined for proteasomal degradation, and the precise target protein within such NCI appears to relate to a non‐ATG‐initiated (RAN) translation of the expanded repeat region which leads to the ‘inappropriate’ formation and aggregation of dipeptide repeat proteins (DPR) within nerve cells of these and other regions [10, 11, 12, 13, 14].

Most of the early pathological descriptions following the discovery of the *C9ORF72* mutation indicated that the associated TDP‐43 pathology was usually type B, characterized by NCI in all cortical layers but with relatively few dystrophic neurites or neuronal intranuclear inclusions [7, 8, 9, 15]. However, more recent studies have frequently reported a significant proportion of *C9ORF72* mutation cases to have other patterns of FTLD‐TDP, most often type A [9, 11, 15], and rarely type C [16]. Nonetheless, all cases bearing expansions appear to show a similar and characteristic DPR pathology irrespective of what TDP‐43 histological type may also be present [13, 14].

In the present study, we have investigated 22 patients from the Newcastle cohort with pathologically confirmed FTLD‐TDP, and have detected three patients bearing a hexanucleotide repeat expansion in *C9ORF72*. These three patients died prematurely, two from bronchopneumonia within 10 months and 3 years of onset, and one from myocardial infarction 3 years after onset. In all of these, there were abundant DPR within small interneurones and some pyramidal cells throughout cerebral cortical regions and hippocampus and especially so within granule cells of the cerebellum, but TDP‐43 pathological changes, were absent or extremely sparse. Comparisons of TDP‐43 and DPR pathology in these three individuals with others from a Manchester series where both TDP‐43 and DPR pathology were robustly present suggest that in patients with FTLD bearing expansion in *C9ORF72*, widespread accumulation of DPR within nerve cells may occur much earlier than that of TDP‐43.

## Methods

### Patients

Brain tissues were available in the Newcastle Brain Tissue Resource (NBTR) from a series of 22 patients with FTLD (cases #1–22, 12 men, 10 women) from the North East of England. In addition brains were also available from the Manchester Brain Bank (MBB) from 13 other patients with FTLD (cases #23–35, eight men, five women) known to bear expansions in *C9ORF72* [11, 13] from the North West of England and North Wales (supplementary Table S1). All 35 patients fulfilled Lund‐Manchester clinical diagnostic criteria for FTLD [2, 17]. All brains had been obtained with full ethical permission following consent by the next of kin. Ethical permission for this study was granted by NBTR and MBB under their Generic Tissue Bank Approval (reference numbers 08/H0906/136 and 09/H0906/52 respectively) from Newcastle and North Tyneside 1 REC following peer review by their respective Brain Bank Management Panels. Selected clinical and pathological details of these cases have been presented by us elsewhere [15, 18, 19].

### Genetic analysis

The presence of expanded hexanucleotide repeats was determined by repeat primed PCR as described previously [6]. Frozen brain tissue (cerebellum) was available for all 35 cases employed in the study, and Southern blotting was performed as described elsewhere [11]. Expansion sizing was carried out using ImageQuant TL software (Version 7, GE Healthcare) sizing the repeat number against the DIG labelled lambda Hind III labelled size standard included on each gel (Roche Applied Science). Positive control (gDNA isolated from the B‐Lymphocyte cell line ND06769 obtained from the NINDS Repository – Coriell) and negative control were included on each blot, and were required to show a band of the expected size or no signal on hybridization respectively for each blot to pass quality control. Analyses for mutations in *MAPT*, *GRN*, *CHMP2B*, *TARDBP*, *FUS*, *MAPT* haplotype and *APOE* genotype were performed, as reported previously [18, 19].

### Histological methods

Series of paraffin sections were cut (at a thickness of 6 μm) from formalin fixed blocks of representative regions of brain to include (where available) frontal cortex (BA8/9), temporal cortex (BA21/22), cingulate gyrus, insular cortex, motor cortex, inferior parietal and occipital (BA17/18) cortex, posterior hippocampus, basal ganglia (to include caudate nucleus, putamen, globus pallidus and thalamus), mid‐brain (to include substantia nigra and oculomotor nucleus), pons (to include locus caeruleus and V cranial nerve nucleus), medulla (to include inferior olives and XII cranial nerve nucleus), cerebellum (with dentate nucleus) and cervical and lumbar spinal cord. Because some of the cases dated back 20 years or more to their accession date, not all brain regions were now available for study, especially those such as the anterior hippocampus and amygdala, and certain areas within the mid‐brain and brain stem. Spinal cord was only obtained at post‐mortem in a single case. Sections from available areas within the series had been previously immunostained by standardized routine methods for both laboratories (see [18, 19]) for amyloid β protein (Aβ), tau, ubiquitin, TDP‐43 and FUS proteins, employing microwaving in 0.1M citrate buffer, pH 6.0 for antigen retrieval.

Pathologically, of the 22 Newcastle cases, 19 had FTLD‐TDP (nine with type A histology, four with type B histology and six with type C histology), one had FTLD‐FUS (NIFID) and two were pathologically unclassifiable, but were possibly FTLD‐UPS [20]. Among the 13 Manchester *C9ORF72* expansion bearers, eight patients had FTLD‐TDP type A histology, whereas five patients had FTLD‐TDP type B histology [20].

Further sets of sections from those cases within the Newcastle series found on gene analysis to bear an expansion in *C9ORF72*, and all 13 Manchester cases, were all immunostained in the Manchester laboratories for the presence of p62‐immunoreactive NCI by immunostaining with p62‐lck ligand [rabbit polyclonal antibody (B D Biosciences, Oxford, UK) 1:100] employing a standard ABC Elite kit (Vector, Burlingame, CA, USA) with DAB as chromogen, and again microwaving in 0.1M citrate buffer, pH 6.0 for antigen retrieval. Positive cases were defined where p62‐positive, TDP‐43‐negative NCI within either the cerebellum (see [8]) or hippocampus (see [7]) could be clearly seen under low power objective (×20) and the majority of high power fields (×40) contained at least two NCI. Either negative cases were completely devoid of p62 immunostaining, or small amounts of apparently extracellular and ‘extraneous’ p62‐positive particulate material was observed in occasional high power fields. Another set of sections from these same cases from both series was immunostained for DPR (again in Manchester) using a polyclonal poly‐GA antibody (courtesy of Dr M Hasegawa) as described elsewhere [11, 13].

### Pathological assessment

The presence of DPR immunostained NCI within nerve cells was assessed at ×20 magnification in those brain regions where all cases could be represented, according to:

0 = no DPR immunostained NCI present in any field.
0.5 = rare/single DPR immunostained NCI present in entire section.
1 = a few (1–5) DPR immunostained NCI present, in some but not all fields.
2 = a moderate number of DPR (6–10) immunostained NCI present in each field.
3 = many (in excess of 10) DPR immunostained NCI in each field.
4 = very many DPR immunostained NCI present, affecting nearly all cells in every field.

Scores per assessed area were summated across those regions where these were available for all individuals with *C9ORF72* expansions. Brain regions were grouped on an anatomical or a ‘functional’ basis. Hence, scores from frontal, temporal, cingulate, insular, parietal and occipital cortical regions were summated to generate a total ‘cortical’ score for each case. Scores from hippocampus and adjacent regions of subiculum, entorhinal cortex and fusiform gyrus were summated to give a total medial temporal lobe score for each case. Scores in cerebellar granule and Purkinje cells, and those of the dentate nucleus, inferior olives and pontine nuclei, were summated to give a total ‘cerebellar’ score. All scoring of sections was performed by a single rater (DMAM).

The frequency of TDP‐43 pathological changes (as NCI and neurites, where present) in each of frontal and temporal cortex (pyramidal cells of layers II) and hippocampus (dentate gyrus granule cells), was also scored semi‐quantitatively according to the same system employed for DPR pathology. Again, all scoring was performed by a single rater (DMAM).

### Statistical analysis

Rating data were entered into an excel spreadsheet and analysed using Statistical Package for Social Sciences (SPSS) software (version 17.0). Kruskal–Wallis with *post‐hoc* Mann–Whitney test was used to compare inclusion scores between several groups, or Mann–Whitney alone for pairs of groups. anova with *post‐hoc* Tukey test was used to compare ages at onset and death, and duration of illness, between several groups. In all instances, a *P*‐value of less than 0.05 was considered statistically significant.

## Results

### Genetics

Of the 22 Newcastle cases investigated, three (patients 1–3 described below) demonstrated an expansion in *C9ORF72* by both repeated primed PCR [6] and by Southern blotting [11]. The 13 Manchester patients with FTLD, known to bear expansions in *C9ORF72*, have been reported elsewhere [11, 13]. In all 16 expansion carriers, expansion size ranged from ∼5 kb (∼450 repeats) to in excess of 23 kb over 3600 repeats). No pathological variants of *GRN*, *CHMP2B*, *TARDBP*, *FUS*, *MAPT* haplotype and *APOE* alleles were found [18, 19].

### Case histories

Case histories for the three Newcastle patients bearing expansion in *C9ORF72* are presented below. Clinical details on the Manchester patients have already been presented elsewhere [15].

#### Newcastle patient 1

This 63‐year‐old man presented in January 1995 with coryzal symptoms and persistent headache, followed by an overnight change in his personality (withdrawal, altered sleep pattern and features of catatonia). CSF studies, imaging and EEG did not reveal any clear abnormality. In the past he had a small myocardial infarction (EKG confirmed). Clinically, encephalitis lethargica was considered, but he did not respond to anticholinergic medication, and this diagnosis was never definitely established. His condition deteriorated while a hospital inpatient, becoming bedbound, doubly incontinent with pressure sores. Agitation was treated with morphine. He died 10 months later from bronchopneumonia.

#### Newcastle patient 2

This 73‐year‐old woman presented with a 1‐year history of memory loss and 2 months loss of appetite. There was a history of TIA at the age of 71. On examination, she was quiet, pleasant and co‐operative and fully orientated. She had nonfluent speech with receptive and expressive difficulties, mild naming difficulties and abnormal prosodic output. Her affect was flat, but she was responsive to humour. There was a mild tongue deviation to the right and increase in limb tone. On the ward she wandered randomly and required treatment including thioridazine, haloperidol and orphenadrine.

At the age of 74 she could not remember where she lived, was disoriented to time, place and person, and made repetitive conversation. Her immediate recall had deteriorated rapidly to 0/5 with poor concentration (3/6). Mental test scores at this time (MTS) [21] varied between of 19.5 to 24 (out of 37). Visual hallucinations were documented once. On examination her tongue still deviated slightly to the right. She had limb hyperreflexia. She died aged 75 years after 3‐year duration of illness. Autopsy confirmed bronchopneumonia with moderate atheroma of the coronary arteries, total atheromatous occlusion of the right – and partial occlusion of the left, carotid artery, and partial constriction at the origin of the vertebral arteries.

#### Newcastle patient 3

This 71‐year‐old man presented with a 3½‐year history of dementia. He had a previous history of myocardial infarction. There was a 20‐month history of fatuous behaviour, neglect, forgetfulness and unsteadiness before death. He became incapacitated, and resistant to washing, dressing and feeding, increasingly stressing his wife. On examination he was scruffily dressed, grunting continuously and inappropriately cheerful. He was fully oriented to place, person and slightly disoriented in time. He was euphoric and displayed disinhibited behaviour, with a mild memory deficit. Neurological examination revealed normal tone and power bilaterally. His gait was unsteady. He required treatment for night‐time wandering with thioridazine. He appeared hyperactive, and forgetful. He was unable to sit down and hid things about the house. He had urinary incontinence. While awaiting a CT scan, he became rapidly short of breath, doubly incontinent and confused. Just before death at the age of 71, he developed abnormal breathing and rhythmical extension movements of the right limbs. Autopsy confirmed an old left anterolateral cardiac infarct.

### General neuropathology

In Newcastle patient 1, brain and spinal cord appeared normal (brain weight 1426 g), whereas in Newcastle patient 2 brain weight was reduced (1230 g) with marked parietal atrophy and moderate frontal, occipital and superior temporal gyrus atrophy (patients 1 and 2 are N19 and N23, respectively, in [18]). Midbrain or brain stem showed no clear macroscopical evidence of atrophy in any of the patients. In Newcastle patient 3 (patient N24 in [18]), actual weight was not recorded, but the brain showed mild frontotemporal atrophy. The extent of neurodegeneration (as exemplified by apparent nerve cell loss and cortical microvaculation) in upper layers of frontal, temporal and parietal cortices was only mild to moderate in all three patients. Patient 1 showed no pathological evidence of encephalitis and loss of cells from substantia nigra was mild. There was, however, severe loss of neurones from areas CA1–4 with hippocampal sclerosis, whereas in patients 2 and 3 the CA1 sector showed only mild to moderate neuronal loss and hippocampal sclerosis could not be definitely ascribed. All three patients showed a mild Purkinje cell loss and mild loss of neurones from the dentate nucleus, with a few silver‐positive tangles being present in the latter region in patient 3. Patient 2 showed a subtotal neuronal loss in the hypoglossal nucleus on one side, although no TDP‐43‐positive inclusions were seen. In patient 3, the subcortical white matter showed considerable myelin pallor, this extending into the midbrain, cerebellum and upper cervical cord. General neuropathological changes in the Manchester series of cases have already been presented by us elsewhere [15, 18].

Immunostaining for tau showed (usually only scant) AT8‐positive neurones only in patient 3 of the Newcastle cases, and in 9/13 (70%) Manchester cases, with *C9ORF72* expansion. AT8‐positive neurones were located mostly in the hippocampus (CA1 and/or subiculum), but also occasionally within pyramidal cells in inferior and middle temporal gyri. None of the cases achieved a Braak score of more than II, usually less. Immunostaining for Aβ showed occasional plaques, again in patient 3 of the Newcastle cases, and in 3/13 (23%) Manchester cases, with *C9ORF72* expansion. These were generally present as diffuse amyloid deposits, mostly within the medial temporal cortical regions, and received CERAD score of A. Immunostainings for alpha‐synuclein and FUS were negative for all cases.

### TDP‐43 immunostaining

**Figure 1:**
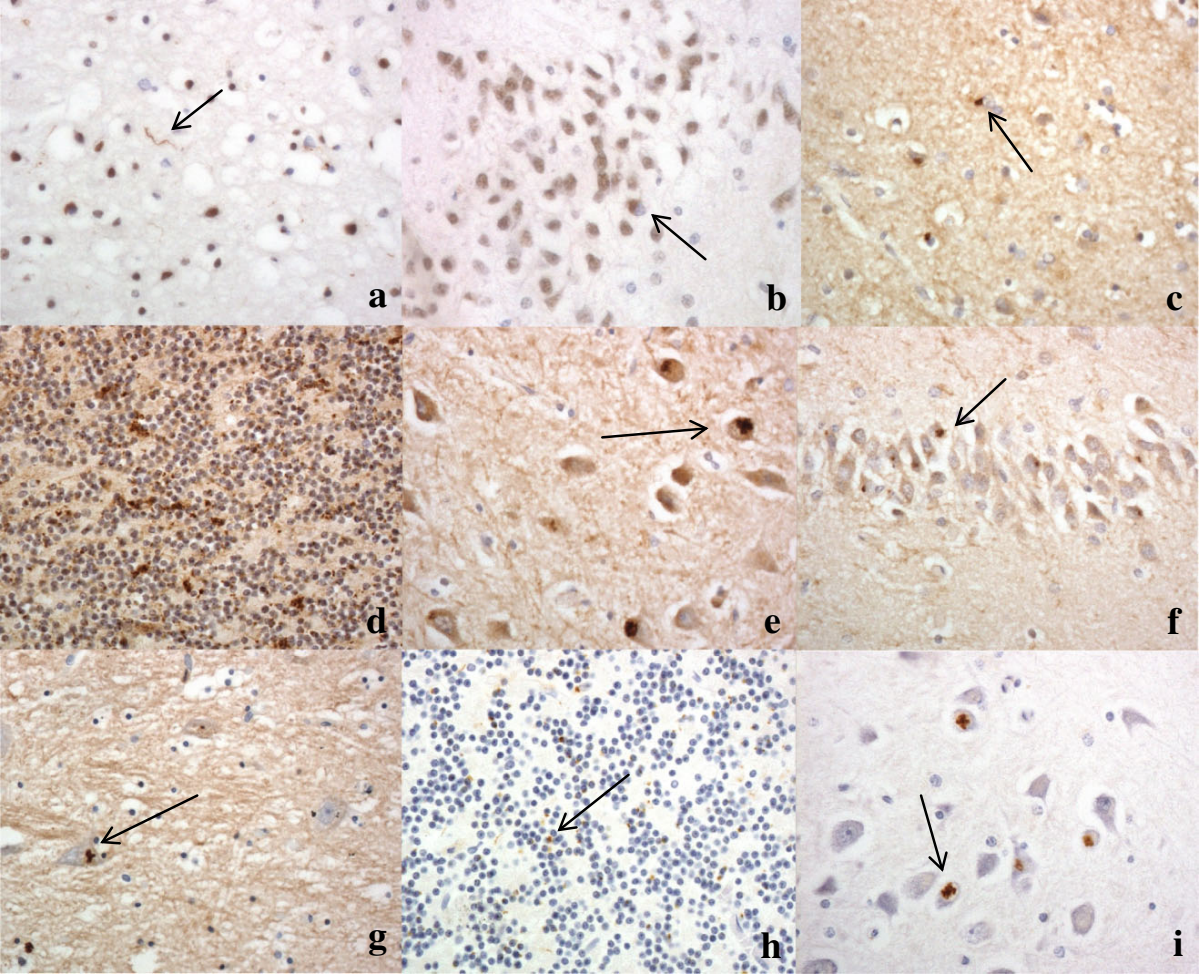
Sparse TDP‐43 pathological changes in case 2, seen as occasional neurites in the cerebral cortex (**a**), and rare neuronal cytoplasmic inclusions in neurones of the dentate gyrus of the hippocampus (**b**). Poly‐GA immunostaining shows moderate to many neuronal cytoplasmic inclusions in small neurones of the temporal cortex (**c**), cerebellar granule cells (**d**), neurones of CA4 region (**e**) and granule cells of dentate gyrus (**f**) of the hippocampus and neurones of the ventrolateral nucleus of the thalamus (**g**). Immunostaining for p62 protein shows relatively fewer neuronal cytoplasmic inclusions in cerebellar granule cells (**h**) and CA4 pyramidal cells of the hippocampus (**i**) compared with poly‐GA immunostaining (compare with (**d)** and (**e**) respectively). Immunoperoxidase‐haematoxylin. ×40 microscope objective magnification.

Newcastle patients 1 and 2 showed very sparse TDP‐43‐positive neurites, but no NCI, in frontal and temporal lobes (Figure 1 **a**), and only an occasional (usually only a single) TDP‐43‐positive, but FUS‐negative, NCI in the dentate gyrus (Figure 1 **b**; Tables 1 and 2). A pathological diagnosis of FTLD‐TDP, suggestive of type A [20], was ascribed to both. Newcastle patient 3 showed a few ubiquitin/TDP‐43‐positive NCI in the dentate gyrus, and in frontal and temporal lobes, with rare scattered neurites in these latter regions; a pathological diagnosis of FTLD‐TDP, suggestive of type B was ascribed (Tables 1 and 2). No TDP‐43 NCI or neurites were seen in any other brain region in any of the three cases. The 13 Manchester *C9ORF72* expansion bearers all demonstrated a robust TDP‐43 pathology permitting clear histological subtyping: eight patients had type A histology and five patients had type B histology (Supplementary Table S1).

**Table 1:** Clinical and pathological details of the three patients with hexanucleotide repeat expansion in C9ORF72

Patient	Gender	Clinical diagnosis	Pathological diagnosis	Family history	Onse t (years)	Duration (months)	Brain weight (g)
1	M	Encephalitis lethargica	FTLD‐TDP Possibly type A	na	63	10	1426
2	F	Vascular dementia/atypical AD*	FTLD‐TDP Probably type A	Sister had ‘stroke’, was dysarthric	72	36	1230
3	M	Vascular dementia*	FTLD‐TDP type B	na	68	36	na

*Blinded retrospective clinical impressions were achieved by the review of relevant clinical histories in these patients.

FTLD, frontotemporal lobar degeneration; AD, Alzheimer's disease; na, not available.

**Table 2:** Relative brain distribution of poly‐GA immunopositive neuronal cytoplasmic inclusions (dipeptide repeat proteins, DPR), and TDP‐43 immunopositive neuronal cytoplasmic inclusions and/or neurites in the three Newcastle cases of frontotemporal lobar degeneration (FTLD) bearing hexanucleotide expansions in C9ORF72 gene, and range of scores for DPR and TDP‐43 pathology in the 13 Manchester cases with which these are compared

Brain region	Newcastle case 1		Newcastle case 2		Newcastle case 3		Manchester cases	
	DPR	TDP	DPR	TDP	DPR	TDP	DPR	TDP
Frontal cortex	3	0.5	1	0.5	2	0.5	2–4	1–3
Temporal cortex	3	0.5	2	0.5	2	1	2–4	1–3
Cingulate cortex	2	0.5	1	0.5	2	0.5	2–4	1–3
Insular cortex	3	0	1	0	2	0	2–4	0
Entorhinal cortex	2	0	2	0	0	0	2–4	0
Fusiform gyrus	2	0	2	0	1	0	2–4	0
Parietal cortex	2	0	1	0	2	0	2–4	0
Occipital cortex	1	0	2	0	1	0	2–4	0
Hippocampus dentate gyrus	2	0.5	2	0.5	2	0.5	2–4	1–3
Hippocampus CA4 region	2	0	3	0	2	0	2–4	0
Hippocampus CA3 region	2	0	2	0	1	0	2–3	0
Hippocampus CA2 region	0.5	0	1	0	1	0	1–2	0
Putamen	0.5	0	0	0	na	na	0–1	0
Thalamus	1	0	na	na	na	na	1–3	0
Cerebellar granule cells	4	0	3	0	4	0	3–4	0
Purkinje cells	0	0	0	0	0.5	0	0–1	0
Dentate nucleus	0.5	0	0	0	0	0	0–1	0

na, tissue not available.

### DPR immunostaining

All 16 cases with expansions in *C9ORF72* showed qualitatively similar changes with respect to DPR pathology, although the extent of such changes varied between cases and brain regions. These are illustrated in Figure 1 (and Table 2) with reference to the three Newcastle cases, although similar changes were seen in the Manchester cohort, and these have been described and presented elsewhere (see supplementary Figure S1 and [11, 13]).

For all three Newcastle cases, sections of cerebral cortex immunostained for DPR with poly‐GA antibody showed small clusters of immunostained granules within the cytoplasm of mostly small interneurones within layers II and IV, particularly (Figure 1 **c**). Less commonly, NCI with a more spicular or star‐shaped appearance were seen within pyramidal neurones, mostly those in layers V and VI. Regionally, these were widespread throughout all neocortical regions, though appeared to be more common within frontal, cingulate, insular and temporal cortex than, entorhinal, parietal and occipital cortex (Table 2).

Sections of cerebellar cortex immunostained for DPR with poly‐GA antibody showed small rounded or oat‐shaped NCI within granule cells, though occasionally larger, more rounded and solid NCI (as described in reference [8]) were seen (Figure 1 **d**). A few, thin and short neuritic profiles (DN) were seen, and neuronal intranuclear inclusions (NII) were occasionally observed. These were very common in patients 1 and 3, but slightly less plentiful in patient 2 (Table 2). Similar, more granular, NCI were usually present in basket cells, but none were seen within Golgi neurones, or within Bergmann glia. Occasional Purkinje cells contained small, spicular or granular p62‐immunoreactive structures, but none were seen in neurones of the dentate nucleus.

In the hippocampus, rounded or granular p62‐immunoreactive NCI were present in dentate gyrus granule cells (Figure 1 **e**), and NCI were commonly seen in pyramidal cells of CA4 and CA3 regions of the hippocampus, where these had a spicular or star‐shaped appearance (as described in reference [7]) (Figure 1 **f**), but were less common in CA2, and absent in CA1 and subiculum.

Generally, DPR were sparse within basal ganglia regions. They were occasionally present, in case 1 alone, as spicular NCI in small neurones in the putamen, and in larger neurones of the ventrolateral nuclei (Figure 1 **g**, Table 2), but were not seen in cells of the substantia nigra, locus caeruleus, nucleus basalis of Meynert, dorsal raphe nucleus, basalis pontis, inferior olives, motor nuclei of III, IV, V, X or XII cranial nerves, or in anterior horn cells of the spinal cord.

### p62 immunostaining

In all brain regions investigated, the DPR immunoreactive NCI were also immunoreactive to p62, although often the frequency of p62‐positive inclusions appeared less than that seen by DPR immunostaining (Figures 1 **h,i**). No p62/DPR pathology was seen in the other FTLD cases (16 with FTLD‐TDP) not bearing expanded hexanucleotide repeats.

### Comparisons between the three Newcastle and 13 Manchester cases

TDP‐43 and DPR scores for the three Newcastle patients and the range of scores for the 13 Manchester patients are shown in Table 2. Scores for TDP‐43 pathology, and summated regional scores for DPR pathology in the individual Manchester patients are presented in supplementary Table S2.

Comparisons of the extent of DPR and TDP‐43 pathology were made between the three Newcastle cases with early termination of illness, and the 13 Manchester cases with established, end‐stage disease. However, because, the Newcastle cases had a short duration of illness (up to 3 years) (mean duration 2.3 ± 1.3 years), further comparisons were also made between these cases and the five Manchester cases where duration of illness was less than 4 years (mean duration 2.4 ± 0.9 years) and the other eight Manchester cases where duration of illness was greater than 5 years (mean duration 8.6 ± 4.4 years) (anova test, *F*_2,13_ = 7.30, *P* = 0.007 for comparisons of disease duration between the three groups), with the mean duration of illness being significantly longer (by Tukey test) in the eight longer‐duration Manchester cases compared with both the three Newcastle cases and the five shorter‐duration Manchester cases (*P* = 0.034 and *P* = 0.014 respectively), which in turn did not differ from each other (*P* = 0.998). Mean age at onset and age at death did not differ between the three Newcastle cases, and the shorter and longer‐duration Manchester cases (*F*_2,13_ = 2.4, *P* = 0.130 and *F*_2,13_ = 0.28, *P* = 0.758 respectively) (Table 3).

**Table 3:** Mean ± SD age at onset, age at death and duration of illness for the three Newcastle cases with expansion in C9ORF72, and the 13 Manchester cases, overall, and stratified into those with shorter (cases 23–27) and longer (28–35) disease durations

Group	Age at onset (years)	Age at death (years)	Duration of illness (years)
Newcastle cases 1–3	67.7 ± 4.5	70.0 ± 5.6	2.3 ± 1.3
Manchester cases 23–27	64.2 ± 8.1	66.6 ± 8.9	2.4 ± 0.9
Manchester cases 28–35	58.5 ± 6.4	67.1 ± 4.9	8.6 ± 4.4
Manchester cases 23–35	60.7 ± 7.4	66.9 ± 6.4	6.2 ± 4.6

The severity of TDP‐43 pathological changes was significantly less in the three Newcastle cases compared with all 13 Manchester cases for scores for dentate gyrus (*P* = 0.018), temporal cortex (*P* = 0.013) and frontal cortex (*P* = 0.007). Comparing scores for the three Newcastle cases, the five Manchester shorter‐duration cases and the eight Manchester longer‐duration cases (by Kruskal–Wallis test) also showed significant differences between the groups for dentate gyrus (χ^2^ = 5.7, *P* = 0.050), temporal cortex (χ^2^ = 6.1, *P* = 0.046) and frontal cortex (χ^2^ = 7.7, *P* = 0.021). *Post‐hoc* (Mann–Whitney) tests showed significant differences in TDP‐43 pathological scores between the three Newcastle cases and the five shorter‐duration Manchester cases for dentate gyrus (*P* = 0.035), temporal cortex (*P* = 0.034) and frontal cortex (*P* = 0.034), and between the three Newcastle cases and the eight longer‐duration Manchester cases for dentate gyrus (*P* = 0.022), temporal cortex (*P* = 0.022) and frontal cortex (*P* = 0.007). However, there were no significant differences in TDP‐43 pathological scores in any region between the five shorter‐, and the eight longer‐, duration Manchester cases.

However, comparing DPR pathological scores showed no significant differences between the severity of DPR pathology between the three Newcastle cases and all 13 Manchester cases for cortical (*P* = 0.191), medial temporal (*P* = 0176) or cerebellar (*P* = 0.635) scores. Comparing DPR scores for the three Newcastle cases, the five Manchester shorter‐duration cases and the eight Manchester longer‐duration cases (by Kruskal–Wallis test) also showed no significant differences in scores between the three groups for cortical (χ^2^ = 2.7, *P* = 0.258), medial temporal (χ^2^ = 3.1, *P* = 0.212) or cerebellar (χ^2^ = 4.35, *P* = 0.113) scores.

## Discussion

In the present study, we have detected three patients from the Newcastle FTLD cohort of 22 patients with FTLD with proven expansion in *C9ORF72* who died prematurely due to incidental illness in what is considered to be early pathological stages of their disease course. Although, their clinical features were ascertained retrospectively from clinical records, and as such should be treated with some caution, they do seem to show a fairly rapid cognitive decline in common. Two of these patients (patients 2 and 3) can clearly be regarded as late onset FTLD [19]. Patient 1 deteriorated rapidly and died after 10‐month duration of illness, without extensive clinical or neuropsychological follow‐up, although clinical observation led to the impression of FTLD. Patient 2 had motor signs, but the presence of any features of MND [22] was not established robustly, either clinically or pathologically. She died after 3‐year illness. She also had a progressive aphasia and psychosis. Psychosis is generally rare in FTLD, but has recently been emphasized as a characterizing feature in FTLD patients with *C9ORF72* mutation [15], especially those with associated MND [23].

The extent of DPR and TDP‐43 pathological changes in these three patients was compared with those seen in pathologically more established cases of FTLD with *C9ORF72* expansions dying with end‐stage disease [13, 14]. All three cases showed characteristically abundant p62‐ and poly‐GA immunopositive NCI widespread within the cerebral cortex, cerebellum and hippocampus, in a topographical, as well as numerical, distribution similar to the established cases. However, in two cases (patients 1 and 2) there was only rare TDP‐43‐positive NCI within the hippocampus dentate gyrus, and very sparse TDP‐43 immunopositive pathological changes, mostly as short neurites with only rare, or no, NCI, within frontal and temporal cortex. These two cases were diagnosed with FTLD‐TDP, suggestive of type A [20]. Another case (patient 3) bore more abundant TDP‐43 immunopositive NCI in hippocampus and cortex and conformed to FTLD‐TDP type B [20]. The extent of TDP‐43 pathology (and neurodegeneration as evidenced by cortical neuronal loss and microvacuolation) in all three patients was considerably less than that generally encountered in established late cases of FTLD with *C9ORF72* expansion [15], supporting the view that these particular cases were indeed in the early stages of their illness when they died.

When scores for TDP‐43 pathology were compared, it was seen that the severity of TDP‐43 pathological changes was significantly less in the three Newcastle cases than that present in the Manchester cases, even when those cases were stratified to match length of disease duration to the Newcastle cases. On the other hand, DPR pathological changes were similar in extent in the Newcastle cases to those of the Manchester series, even when stratification for duration of illness was considered. Hence, semiquantitative comparisons of TDP‐43 and DPR pathological changes bear out the microscopic impressions that the three Newcastle cases had indeed died in the early pathological stages of the disease, at least as far as TDP‐43 pathology is concerned, even though two of these patients had suffered duration of illness similar to some of those in the Manchester series with shorter durations, yet had not developed an equivalent level of TDP‐43 pathology. Conversely, the extent of DPR changes in these three cases was similar to those Manchester cases where TDP‐43 pathology was more robustly established, even in those with equivalent durations of illness.

These three patients, and especially patients 1 and 2, are therefore of special interest as while there was abundant DPR pathology in the cerebral cortex, hippocampus and cerebellum, TDP‐43 pathology was generally sparse with only rare NCI, and few neurites, being present. Such observations are reminiscent of a recent report [24] in which a family bearing expansion in *C9ORF72* and demonstrating intellectual disability as a preceding feature to dementia at an early age is described. In this family, the proband presented with psychiatric features progressing into typical FTD and at autopsy showed extensive DPR, but only minimal TDP, pathology. Indeed, the latter was so sparse as to be initially overlooked. In her intellectually disabled son, dying at age 26 years, also with expansion in *C9ORF72* only DPR aggregates were found, and no TDP‐43 changes whatsoever. Together such findings suggest that deposition of DPR aggregates within the brain in patients bearing expansions in *C9ORF72* may precede that of TDP‐43 pathology.

The most straightforward, and logical, conclusion of such observations is that formation of DPR throughout many brain regions may be an early/the earliest event in the pathogenesis of FTLD associated with expansions in *C9ORF72*, and that in some way this leads to or predisposes towards the subsequent development of TDP‐43 proteinopathy. However, the mechanism by which this might take place remains obscure. Indeed, there are good reasons for arguing that there is in fact no direct link between the two pathologies. First, studies on patients bearing expansions in *C9ORF72* with established DPR and TDP‐43 pathologies have shown that essentially the two aggregating proteins are contained within separate neuronal populations [7, 8, 9, 10, 11, 12, 13, 14] with only occasional colocalization [14]. Indeed, on the rare occasions this does occur it would appear that TDP‐43 is secondarily deposited upon pre‐existing DPR [14]. Second, despite there being reports of cerebellar atrophy in some cases with *C9ORF72* expansions [25, 26], the presence of p62/DPR histological changes in this region appear clinically silent, at least when assessed using conventional measures of cerebellar function. This is despite present observations that DPR pathology appeared heavier within cerebellum than either cerebral cortex or hippocampus at early stages of the illness. Third, TDP‐43 pathology is never encountered within many brain regions vulnerable to DPR pathology besides the cerebellum, such as parietal and occipital cortex, CA2/3/4 neurones of hippocampus and cells of ventrolateral nucleus of the thalamus [11]. Fourth, we have noted no histological distinctions between FTLD‐TDP type A or type B cases carrying expansions in *C9ORF72* from those without expansions, other than the presence of p62/DPR in the former [11]. Lastly, the occurrence of several cases of FTLD where an expansion in *C9ORF72* is seen in association with either *MAPT* [27, 28] or *GRN* [28] mutation suggests this is not likely to be pure chance, and in such cases, either tauopathy or FTLD‐TDP type A histology, typical of the accompanying mutation is present along with the DPR pathology.

Collectively, these observations imply that while the accumulation of DPR is a pathological feature of the expansion in *C9ORF72*, and indeed can act as a tissue marker of its presence, there is no good evidence that DPR are pathogenic *in vivo*. Nonetheless, it remains possible that the expansion in *C9ORF72* may act as a ‘gatekeeper’, ‘weakening’ the brain, in a so far mechanistically undiscovered way, thereby allowing or facilitating the development of ‘sporadic’ disease, or promoting other genetic forms of FTLD associated with TDP‐43 [28], or even tau [27, 28], pathology to develop. In such a scenario, there would be no requirement to directly link DPR changes associated with the expansion to those causing TDP‐43 pathology. DPR pathology would represent a kind of innocent by‐stander which, while of diagnostic importance, would remain pathogenically benign.

## Author contributions

David Mann and Atik Baborie provided study design, performed all microscopical assessments, data analysis and wrote the paper.

Atik Baborie, Timothy Griffiths, Evelyn Jaros, Robert Perry, Ian McKeith and David Burn provided clinical data and neuropathological characterization of the Newcastle cases.

Yvonne Davidson and Andrew Robinson prepared sections for staining and performed the immunohistochemistry.

Andrew Robinson helped with statistical advice and data analysis.

Sara Rollinson and Stuart Pickering‐Brown performed the genetic analyses.

Masami Masuda‐Suzukake and Masato Hasegawa prepared and characterized the DPR antibody.

## Supporting information


**Figure S1.** Topographic brain distribution of dipeptide repeat proteins (poly‐GA) in patients with established FTLD associated with an expansion in *C9ORF72*. Regions shown are frontal cortex layer II (**a**), frontal cortex layer V (**b**), dentate gyrus (**c**) and area CA4 (**d**) of hippocampus, ventrolateral nucleus of thalamus (**e**), granule cells (**f**) and Purkinje cells (**g**) of cerebellum, dentate nucleus (**h**) and putamen (**i**). Immunoperoxidase‐haematoxylin ×40 microscope magnification.

**Table S1.** Selected case details of 22 Newcastle, and 13 Manchester, patients with FTLD.

**Table S2.** TDP‐43 and composite DPR scores for individual patients with expansions in *C9ORF72*.
