# Supplementary figures and images for: Accumulation of dipeptide repeat proteins predates that of TDP‐43 in frontotemporal lobar degeneration associated with hexanucleotide repeat expansions in C9ORF72 gene
Source: Neuropathol Appl Neurobiol. 2015 Apr 30;41(5):601–12. doi: 10.1111/nan.12178 (PMC4934135; doi:10.1111/nan.12178)

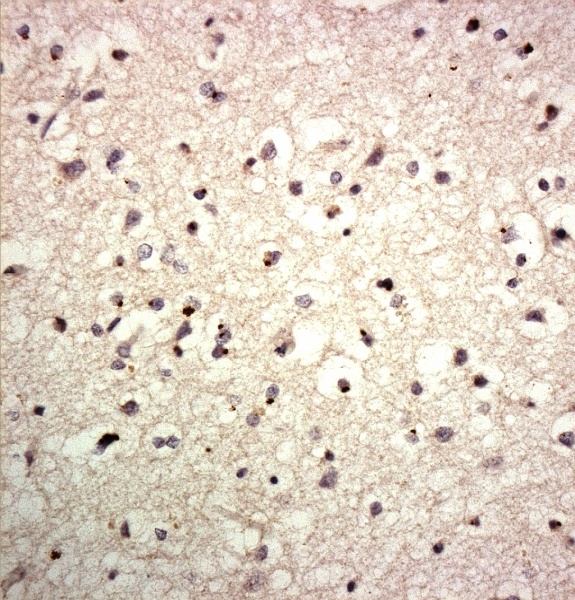

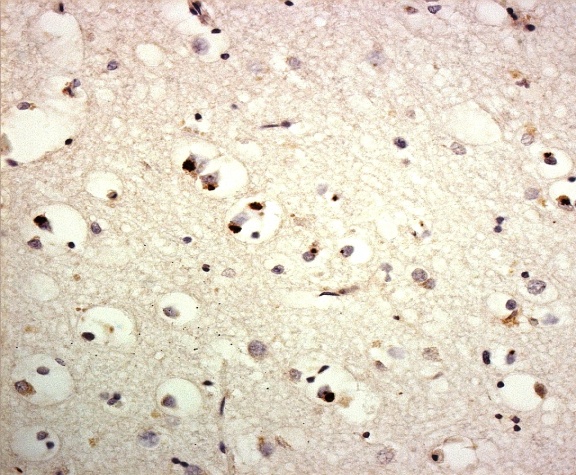


**c**

**b**

**a**


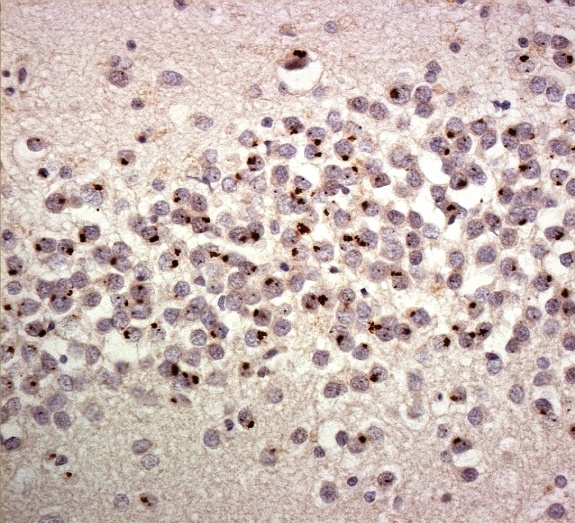

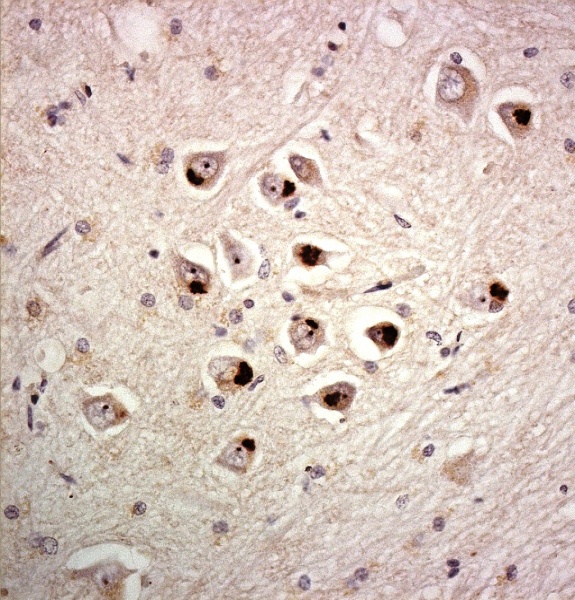


**d**


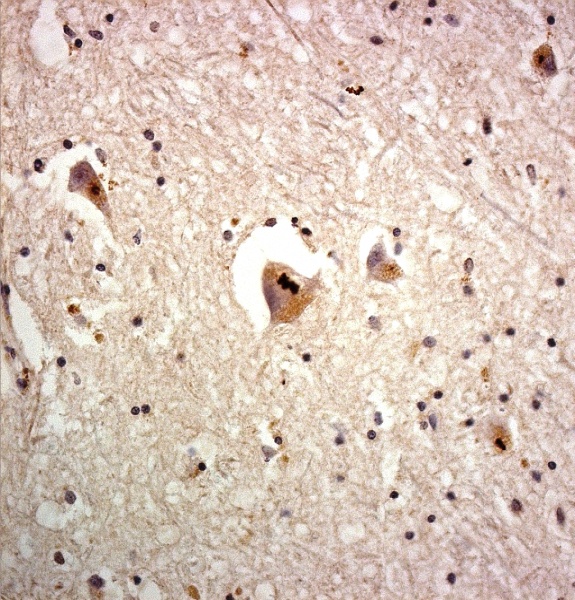

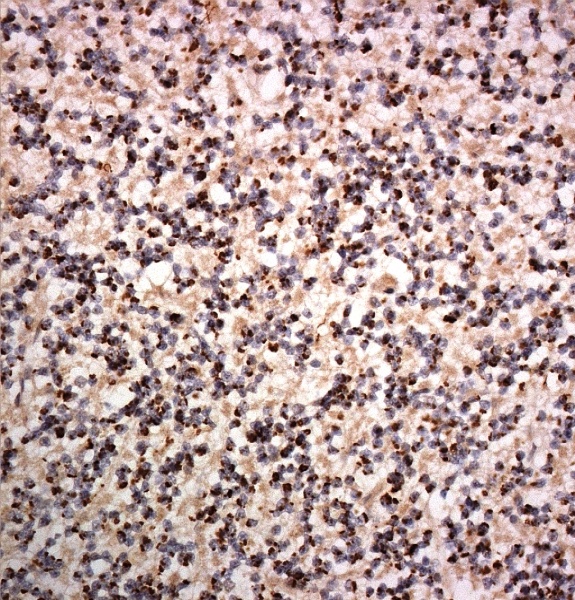


**f**

**e**


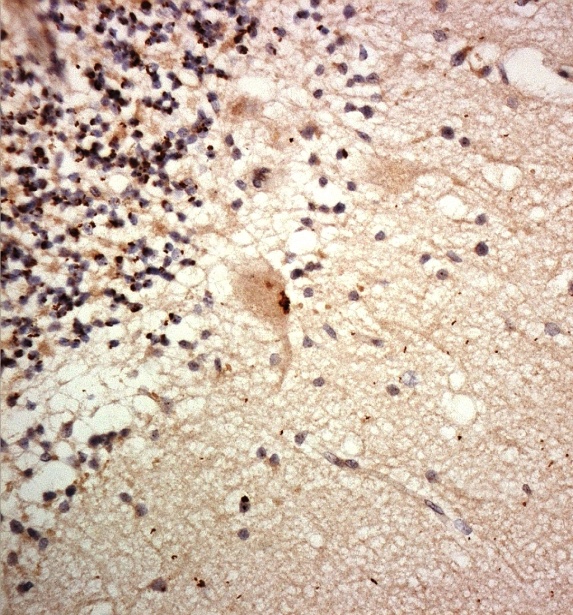

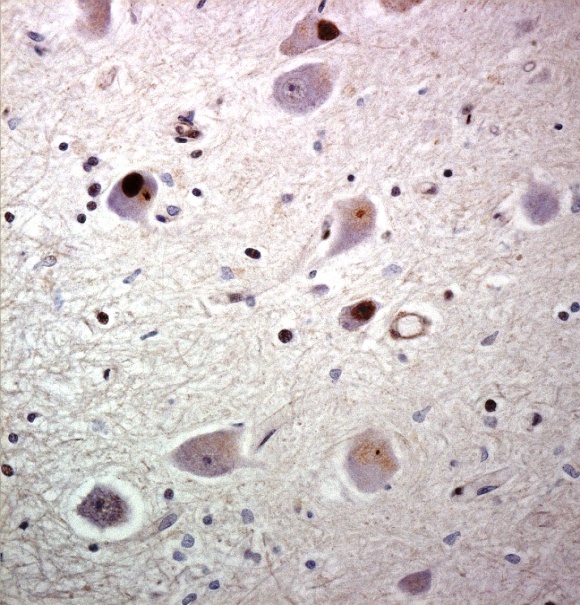


**h**

**g**

Supplement: Supplementary file 1 — Figure S1. Topographic brain distribution of dipeptide repeat proteins (poly‐GA) in patients with established FTLD associated with an expansion in C9ORF72. Regions shown are frontal cortex layer II (a), frontal cortex layer V (b), dentate gyrus (c) and area CA4 (d) of hippocampus, ventrolateral nucleus of thalamus (e), granule cells (f) and Purkinje cells (g) of cerebellum, dentate nucleus (h) and putamen (i). Immunoperoxidase‐haematoxylin ×40 microscope magnification. [file NAN-41-601-s001.docx]
